# microRNA expression in the aging mouse lung

**DOI:** 10.1186/1471-2164-8-172

**Published:** 2007-06-15

**Authors:** Andrew E Williams, Mark M Perry, Sterghios A Moschos, Mark A Lindsay

**Affiliations:** 1 Biopharmaceutics Research Group, Airway Disease, National Heart and Lung Institute, Imperial College, London, SW3 6LY, UK,

## Abstract

**Background:**

MicroRNAs (miRNAs) are a novel class of short double stranded RNA that mediate the post-transcriptional regulation of gene expression. Previous studies have implicated changes in miRNA expression in the regulation of development and the induction of diseases such as cancer. However, although miRNAs have been implicated in the process of aging in *C. elegans*, nothing is known of their role in mammalian tissues.

**Results:**

To address this question, we have used a highly-sensitive, semi-quantitative RT-PCR based approach to measure the expression profile of 256 of the 493 currently identified miRNAs in the lungs from 6 month (adult) and 18 month (aged) old female BALB/c mice. We show that, despite the characteristic changes in anatomy and gene expression associated with lung aging, there were no significant changes in the expression of 256 miRNAs.

**Conclusion:**

Overall, these results show that miRNA transcription is unchanged during lung aging and suggests that stable expression of miRNAs might instead buffer age related changes in the expression of protein-encoding genes.

## Background

MicroRNAs (miRNAs) are 21–23 nucleotide double-stranded RNA molecules produced from endogenous genes by the sequential action of the RNase III enzymes Drosha and Dicer. At the present time, 493 miRNAs have been identified in mammalian tissues (Sanger Institute miRNA Registry, Release 9.1, February 2007) [1] and shown to regulate the post-transcriptional expression of multiple genes through a mechanism similar to RNA interference (RNAi) [2,3]. Indeed, bioinformatics studies suggest that up to a third of all known genes maybe regulated by this mechanism [4]. Post-transcriptional regulation of gene expression is mediated by the RNA-induced silencing complex (RISC), which uses one strand of the miRNA molecule (the guide strand) to target relevant mRNAs at their 3'-untranslated regions. Once mRNA is targeted by miRNAs, the RISC is thought to inhibit protein production either through blocking translation or by reducing mRNA stability [2,3].

At the present time, the functions of only a fraction of the identified miRNAs are known. However, it appears that miRNAs play an essential role during development, since studies with Dicer knockout mice have shown that these die prematurely and have a variety of developmental abnormalities [5-7]. Furthermore, tissue specific developmental functions of individual miRNAs have been determined in mice and zebrafish. For example, miR-196 expression affected limb development [8], miR-1 and miR-133 cardiogenesis [9,10] and skeletal muscle development [11], and miR-181 enhanced myoblast differentiation [12]. Aberrant miRNA expression has also been implicated in the induction of cancer, particularly within the lung and haematopoietic system. Thus, a number of studies have demonstrated that the development and prognosis of lung cancer is associated with selective changes in the profile of miRNA expression [13,14].

At the present time, nothing is known of the role of miRNAs during aging in mammals, although the aging process has previously been shown to be associated with changes in the transcription of several protein coding genes [15,16]. In contrast, studies in the nematode worm *Caenorhabditis elegans *have shown that alterations in the expression of miRNAs are associated with aging. For example, over-expression of lin-4 led to extended lifespan, while loss of lin-4 resulted in a reduced lifespan [17]. In addition, another study reports an overall age-related decline in miRNA expression [18]. However, it is currently unclear how these findings translate to aging in vertebrate organisms in the context of both senescence and disease.

In previous studies in mouse and human lung, we used a highly sensitive and semi-quantitative RT-PCR array to demonstrate profound changes in the profile of miRNA expression during development [19]. In the present study, we have adopted the same approach to detect changes in lung miRNA expression during aging using inbred BALB/c mice. However, despite the characteristic changes in anatomical structure and decreased expression of the pro-collagen genes we observed no significant alteration in the expression of 256 individual miRNA species. These data imply that the transcription of miRNAs is not influenced by the aging process, which contrasts to the decreased expression in a range of non-miRNA encoding genes.

## Results and discussion

**Figure 1:**
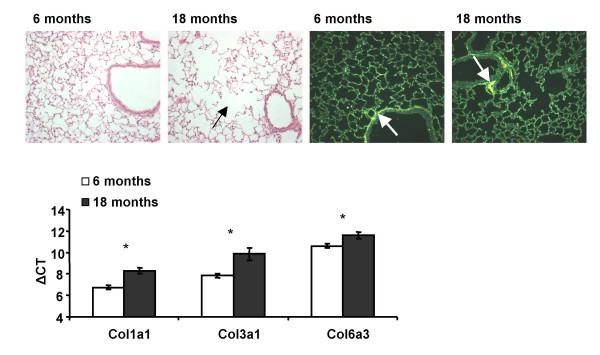
**Morphological changes and gene expression in aged lung tissue**. Histological analysis (haemotoxylin and eosin) of 6 month (A) and 18 month (B) old lung tissue from female BALB/c mice revealed enlarged alveolar ducts and decreased tissue integrity (arrow). Sirius red staining revealed the collagen deposition in adult (C) and aged (D) lung tissue (arrows). Histology was representative of 5 animals from each age group. Analysis of the expression of the protein-coding genes *Col1a1*, *Col3a1 *and *Col6a3 *was performed by RT-PCR (E). Data is represented as the mean ΔCT +/- S.E.M. (CT value of gene – CT value of 18 s), therefore the higher the expression the smaller the ΔCT value. All three genes exhibited statistical significance following 6 months and 18 months comparison, according to Student's t-test (*p < 0.05).

We have previously shown that the miRNA expression profile differs significantly between the fetal and adult lung [19]. In order to determine if similar alterations in miRNA expression occur during aging, we compared adult (6 month old) with aged (18 month old) mouse lung. Structural changes were evident in accordance with aged lung tissue and included enlargement of alveolar spaces associated with a decrease in total surface area (Figure 1A–B). The spatial distribution of collagen was unaltered in the aged lung compared to adult lung, and was most prominent around bronchioles and vasculature (Figure 1C–D). Such structural changes are not necessarily associated with specific diseases *per se*, but are rather more indicative of senescent tissue [20]. Thus, alveolar enlargement and a decrease in surface area are associated with decreased oxygen transfer and arterial oxygen saturation, resulting in the characteristic age related reduction in lung function.

Alterations in collagen and elastin deposition in aged lung tissue have previously been implicated in reduced lung compliance and senescence [21,22]. Therefore the expression of three genes involved in the biosynthesis of collagen were analysed by RT-PCR. The expression of *Col1a1*, *Col3a1 *and *Col6a3 *all significantly decreased in aged lungs compared to adult lungs (Figure 1E). This demonstrated that differences in the expression of conventional protein encoding genes were indicative of a senescent state in 18 month old mouse lung tissue. The role that collagen plays in the aging process and its importance for lung function has sometimes been uncertain. However, our data supports previous studies that show that collagen synthesis is reduced in the aged lung [20,23,24]. The decreased expression of *Col1a1*, *Col3a1 *and *Col6a3 *with age is consistent with the general reduction in the expression of genes involved in biosynthesis [25].

**Figure 2:**
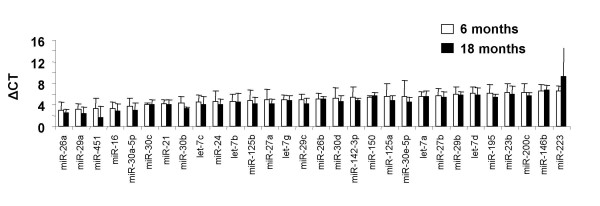
**MicroRNA expression in adult and aged lung**. Semi-quantitative RT-PCR from adult and aged lung tissue revealed a number of miRNAs that were highly expressed irrespective of age. Higher expression is equivalent to a smaller ΔCT value (CT value of miRNA – CT value of 18 s). Statistical significance was measured using Student's t-test (p < 0.05) and data is represented as the mean ΔCT +/- S.E.M. (CT value of gene – CT value of 18 s) of 5 animals.

In order to evaluate whether miRNAs play a significant role in the aging process, a highly sensitive, semi-quantitative RT-PCR method was employed to analyse the expression of 256 individual miRNAs. The relative expression is presented as the ΔCT value for each age group (miRNA CT value – 18 s housekeeping gene value), therefore the smaller the ΔCT value the higher the expression of the miRNA. The expression analysis revealed a number of miRNAs that previously have not been demonstrated to be highly expressed in the lung, including miR-451 (Figure 2). Foregoing research indicated that miR-451 may be involved in the specific differentiation of erythrocytes [26], although such high expression in lung tissue suggests that alternative cell types, such as pulmonary epithelial cells, require miR-451 for their maintenance and/or development. MiR-451 is genomically located at mouse chromosome 11 B5 and the syntenic region at human chromosome 17q11.2, and is expressed in both species from an intergenic region upstream of miR-144 (miR-144 was not available as part of the RT-PCR miRNA expression panel).

In agreement with our previous study [19], miR-26a was the most highly expressed of all the miRNAs profiled in the lung, irrespective of age. The continuing high expression of miR-26a demonstrates that its transcription is unaffected by aging. Such highly expressed miRNAs are likely to play key roles in the maintenance of tissue homeostasis, which is vital for the preservation of normal lung function. It is likely that disregulation of such miRNAs would perturb such homeostasis and may even initiate pathogenesis. Indeed, irregularities in normal miRNA expression have been associated with lung diseases such as cancer [27,28]. It may be the case that miRNA expression is maintained in healthy but senescent tissue and only during diseased states do perturbations in normal expression take place.

**Figure 3:**
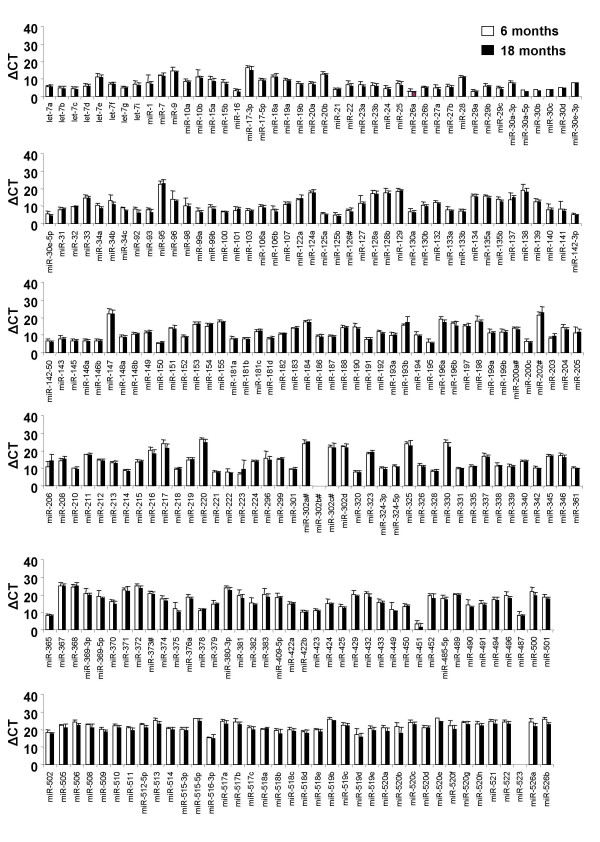
**Comparison of miRNA expression between adult and aged lung tissue**. A highly sensitive RT-PCR panel was used to analyse the expression profile of 256 miRNAs in adult (6 months) and aged (18 months) female BALB/c lung tissue. No significant changes in miRNA expression were observed in aged lung tissue (n = 5 for each age group). Data is represented as the mean ΔCT +/- S.E.M. (CT value of gene – CT value of 18 s). Higher expression is equivalent to a smaller ΔCT value. Statistical significance was measured using Student's t-test (p = 0.05) and data is representative of 5 animals.

Unlike developing lung tissue, there were no significant changes in the expression of any individual miRNA in the aged lung (Figure 3) compared to normal adult lung. These findings are in contrast to the observed changes in mRNA expression in aged tissue [16] and suggest that miRNA encoding genes are transcribed by an independent mechanism. The average ΔCT value for all the miRNAs expressed at 6 months was 13.51 +/- 0.39 and the average ΔCT value at 18 months was 13.04 +/- 0.38. Therefore, no significant alteration in the overall regulation of miRNAs was detected, as normal expression levels were maintained in the aged lung (p = 0.39). These observations also contrast with those in *C. elegans *where a general down-regulation of miRNA expression was observed [18] and suggests that there are species differences. However, it must be remembered that miRNAs involved in aging might still be identified given that new genes are being continually added to miRNA databases and that we examined the expression of 256 of the 493 identified mammalian miRNAs. Furthermore, there might also be differences in miRNA expression that are tissue specific, or that differ between female and male mice. Nevertheless, the majority of recently discovered miRNAs cluster within established miRNA families that were represented in the RT-PCR TaqMan panel [1]. For this reason any changes in the expression of those miRNAs analysed in this study are also likely to represent most of the recently discovered miRNAs.

## Conclusion

MiRNAs are thought to regulate multiple genes and at least a third of all known genes contain miRNA recognition elements. Indeed, the lack of alteration in miRNA expression may buffer age-related changes in the expression of protein-encoding genes. Furthermore, the sustained expression of miRNAs may maintain tissue homeostasis and only in circumstances when miRNA expression is altered, will pathological changes occur.

## Methods

### Animals

Six month old female BALB/c mice (adult) and 18 months old female BALB/c mice (aged) were imported from the National Institute of Aging (National Institute of Health, USA; Harlan Sprague Dawley Inc., USA) and housed in filtered cages according to institutional guidelines and those issued by the Home Office, UK. The average life expectancy of BALB/c females is approximately 20 months. Lungs were removed from animals post mortem and examined for obvious signs of disease. Any diseased tissue was discarded. Lung tissue was either placed immediately in RNA Later (Sigma), or inflated with and placed into 10% neutral buffered formalin (Surgipath).

### Histology

Formalin fixed lung tissue was paraffin embedded and 6 μm sections cut. Sections were either stained with haemotoxylin and eosin or were stained with Sirius red for collagen detection. Haemotoxylin and eosin sections were viewed with normal light microscopy and Sirius red sections with added polarisation. All sections were examined at ×200 magnification for age-associated changes.

### RT-PCR detection of *Col1A1*, *Col3A1*, *Col6A3*

Total RNA was extracted from lungs using miRVana kits (Ambion) according to the manufacturers' instructions and 2 ng/μl was used per RT reaction. The RT reaction contained 0.0775 μl dNTPs, 0.5 μl Multiscribe, 0.75 μl RT buffer, 0.094 μl RNAse inhibitor, 2.081 μl nuclease free water (Promega), 1.5 μl random primers (Applied Biosystems) and 2.5 μl total RNA (2 ng/μl). The reaction conditions were 30 min at 16°C, 30 min at 42°C and 5 min at 85°C. The PCR reaction comprised 12.5 μl TaqMan 2× Universal PCR Master Mix (Applied Biosystems), 8.25 μl RNAse free water (Promega), 1.25 μl 18 s, Col1a1, Col3a1 or Col6a3 probe (Applied Biosystems) plus 3 μl RT product. The reaction conditions were 95°C for 10 min and then 40 cycles of 95°C for 10 seconds and 60°C for 1 min. All samples were normalised to the 18 s housekeeping gene (ΔCT value), and the ΔCT values for each age group compared (the higher the expression the lower the ΔCT value). Statistical significance was demonstrated using a Student's t-test (p = 0.05).

### Semi-quantitative RT-PCR for miRNA expression profiling

The miRNA expression profile for each lung sampled was analysed using Applied Biosystems miRNA TaqMan panel of 256 individual miRNAs. The panel utilises a two step reaction that consisted of reverse transcription primers and separate PCR primers for each miRNA. A total of 2 ng of starting total RNA was used for each miRNA assay. The RT reaction per specific miRNA contained 0.0775 μl dNTPs, 0.5 μl Multiscribe, 0.75 μl RT buffer, 0.094 μl RNAse inhibitor, 2.081 μl nuclease free water (Promega) and 2.5 μl total RNA (2 ng/μl) from TaqMan MicroRNA RT Kit (Applied Biosystems) plus 1.5 μl RT primer (Applied Biosystems) or 1.5 μl random hexamers (Applied Biosystems) and the reaction conditions were 30 min at 16°C, 30 min at 42°C and 5 min at 85°C. The PCR reaction comprised 5 μl TaqMan 2× Universal PCR Master Mix, No AmpErase UNG (Applied Biosystems), 3.835 μl RNAse free water (Promega), 0.5 μl TaqMan probe (Appled Biosystems) or 0.5 μl 18 s probe (Applied Biosystems) plus 0.67 μl RT product and the reaction conditions were 95°C for 10 min and then 40 cycles of 95°C for 10 seconds and 60°C for 1 min. The amount of RNA from each sample was calibrated to the expression of the ribosomal 18 s house-keeping gene. This then gave a delta CT (ΔCT) value for each miRNA (miRNA CT value – 18 s CT value). As 18 s expression was consistently higher than any miRNA, a higher miRNA expression level corresponded to a smaller ΔCT value. Any statistical significance was demonstrated using a Student's t-test (p < 0.05).

## Authors' contributions

AEW coordinated the experiments, performed the microRNA gene expression analysis and drafted the manuscript. MMP and SAM contributed toward the in vivo experiments and assisted in data analysis. MAL participated in the design of the study, assisted in the expression analysis and critically evaluated the manuscript. All authors read and approved the final manuscript.
